# Entrepreneurship, intrapreneurship and scientific mobility: The Spanish case

**DOI:** 10.1371/journal.pone.0201893

**Published:** 2018-09-05

**Authors:** Pedro Aceituno-Aceituno, Joaquín Danvila-del-Valle, Abel González García, Carlos Bousoño-Calzón

**Affiliations:** 1 Department of Business Administration and Management and Economics, Madrid Open University (MOU), Collado Villalba, Madrid, Spain; 2 Department of Business Administration and Management and Economics, Madrid Open University (MOU), Collado Villalba, Madrid, Spain; 3 Department of Criminology, Madrid Open University (MOU), Collado Villalba, Madrid, Spain; 4 Department of Signal Theory and Communications, Carlos III University of Madrid (UC3M), Leganes, Madrid, Spain; IUMPA - Universitat Politecnica de Valencia, SPAIN

## Abstract

Scientific mobility can stimulate entrepreneurship and intrapreneurship, acting as a catalyst for reducing imbalances between local and global science and the resulting socio-economic damage. This study evaluates both whether scientific mobility effectively promotes these concepts and the fundamental reasons to articulate effective policies for scientific mobility. Toward this end, a survey has been prepared following the methodology of *Global Entrepreneurship Monitor* (GEM) and current scientific literature. A total of 364 researchers involved in Spanish scientific mobility took part in the study: Spanish scientists abroad (135) and scientists returned to Spain (52), as mobile groups, and young researchers in Spain (177), as a group of scientists who could go abroad, but that have not yet begun to leave. The results demonstrate that scientific mobility does promote entrepreneurship and, especially intrapreneurship. Moreover, since permanent positions are scarce for these groups and their mobility decisions largely depend on job opportunities, the involved Spanish authorities and agents can improve scientific mobility by means suitable policies that make the most of this potential to the benefit of economic growth and job creation.

## Introduction

International collaboration helps the progress of national research, both if this collaboration is between scientists from their country of origin [1] and, especially, if the collaboration is between researchers in different countries [2–5]. In the latter case, the loss of knowledge may be due to researchers not returning (brain drain), but also it could be due to the elimination or weakening of links with the country of origin (brain circulation) [6]. In order for these relationships to grow and for the countries of origin to be able to access their researchers’ knowledge and their professional networks abroad [7], it is fundamental to maintain intense collaboration with researchers from the country [8, 9], and especially with those from their home institution [10], since there are many cases of researchers returning to their home organizations [11]. However, although these motivating factors for scientific mobility are not only economic [12], this vision of a globalised world in which scientists circulate across various countries carrying out their work and both their countries of origin and their destination countries benefitting from the collaboration, is far removed from the reality of the situation and scientists continue to be attracted by countries that have greater resources [13–15] and offer better salaries [16].

Moreover, mobility has its limitations, since career moves are common but infrequent and generally happen in the early stages of a career [17]. For some countries that are building their research capacity, collaboration can enable them to share others’ progress, but priority might be given not to them but to the national interests of bigger countries, leading to an imbalance between local and global science [18].

In order to achieve greater balance in this area, the influence of government to achieve repatriation is quite limited [19], but it is feasible, since although there is a major difficulty to following up with this group [20, 21] and the main reasons for those who have returned home are personal or family related, the decision of a country’s researchers depends in part on job opportunities [21].

Taking into account these improved job opportunities, the relevance of the concept of cumulative advantages for young researchers is noteworthy, since having these advantages will in future increase the difference between the scientists that have them and those that do not [22]. This concept is called “The Matthew Effect in Science”, taken from the following biblical passage: “For unto everyone that hath shall be given, and he shall have abundance: but from him that hath not shall be taken away even that which he hath^”^ [23, 24].

These advantages are related to the trained capacity, structural location, available resources, institutional prestige and an optimal working environment for world-class teams [25]. Following this important contribution, some later work has highlighted the importance of the scientific career in order to explain their scientific performance [26], especially in the early-stages of their career [27]. In search of these cumulative advantages, researchers are highly competitive and will jump ship in order to find environments that allow them to increase their knowledge, and if they have to move abroad or stay at home in order to achieve this, they are prepared to do so [28].

This international mobility can stimulate these scientists’ entrepreneurship [29, 30], since by working in new, high-tech, business environments they have higher chances to recognise business opportunities [31]. Moreover, this knowledge acquired through international experience allows them access to resources, networks of high entrepreneurial culture, the influence from academic entrepreneurs and to develop these skills, so international mobility improves entrepreneurship [32].

Entrepreneurship is a determinate of the national competitive advantage [33] and generates economic development when innovations make competitors obsolete [34]. Moreover, in our increasingly globalised world, entrepreneurship generates growth, since it acts as a vehicle for innovation as a channel for the diffusion of knowledge to those that are shifting the competitive advantages of modern economies [35].

One of the pillars for ensuring that this economic growth is sustainable is technological innovation [36], since companies with high capacity for growth are those that base their business model on this type of innovation [37]. In general companies with links to scientists establish themselves in high-tech industries such as biotechnology, nanotechnology or semiconductors [38], and although scientists create a relatively small number of companies, these tend to be leaders in terms of innovation and employment [39].

Other authors, such as Yasuda [32] also highlight the fact that scientists create few companies, but their role in carrying out other kinds of entrepreneurial activities within their organizations, such as intrapreneurship, can also be very important. A study by the World Economic Forum (WEF) in collaboration with *Global Monitor Entrepreneurship* (GEM) [40] on the adult population shows that countries with more intrapreneurs in their organizations create more jobs and are more competitive than those with more entrepreneurs. Specifically with regards to competitiveness, each point of increase in this concept is associated with an increase of 2.5% in the rate of intrapreneurs. These rates, defined accordingly to the GEM methodology as seen in the Materials and Methods section, are referred to as Entrepreneurial Employee Activity (EEA). EEA is the proportion of the population aged between 18 and 64 currently involved in and playing leading role in idea development or in the preparation and implementation of a new activity for their employers, such as developing or launching new goods or services or setting up a new business unit or subsidiary. As regards the case of entrepreneurs, the rate used is called Total Early-stage Entrepreneurial Activity (TEA), which is the proportion of the population aged between 18 and 64 either actively trying to start a new business, or managing a business aged less than three and a half years old, in which they have an ownership stake.

For this adult population and according to data from the GEM Report for Spain 2015 [41], the rate of intrapreneurs in Spain (EEA) is 1.1%, placing it 22nd out of 24 countries with innovation-based economies. Likewise, the level of entrepreneurs is low (TEA) with 5.7% and 21st place among innovation-based economies. Of this percentage, in the same vein as the above figures, almost 7 out of every 10 new companies are not focused on innovation and 8 of every 10 do not have an international outlook in the first 3–4 years of business. Likewise, in this GEM Spain Report 2015 report there are no data on groups involved in scientific mobility, the only data given for groups relates to entrepreneurs with postgraduate training (Masters or Doctorate), which again highlights that these groups are among those that create the fewest companies, with a TEA of 7.8% of the total percentage.

As far as the scientists’ activities within companies is concerned, Spain is one of the developed countries with the lowest percentage of researchers in companies, with a percentage that has ranged over the past ten years between 34.5% and 36.9% [42] well below the average for the EU-27 (49.0%), the United States (79.0%) or Japan (68.0%) [43]. These figures show that the business culture is possibly not very well-rooted with Spanish scientists.

On the other hand, different studies carried out since the start of the decade until now show that agents of the *Sistema Español de Ciencia y Tecnología* (SECTI) can influence the mobility of scientists, since this largely depends on job opportunities and the scientific career [44–46]. Likewise, with regards to the possibility of these agents collaborating with Spanish scientists abroad, the level of collaboration between these scientists and international institutions is quite high, but much reduced with national scientific institutions and the home institution in Spain of each researcher [47].

In view of the foregoing, this work aims to provide data on the entrepreneurship and intrapreneurship of Spanish scientific mobility, to assess whether this mobility effectively promotes this entrepreneurship and intrapreneurship and to discover the fundamental reasons for this effectivity, in order to enable the development of effective policies for scientific mobility that stimulate entrepreneurship and intrapreneurship, favouring economic growth, job creation and the right balance between local and global science.

## Materials and methods

This study includes three groups of Spanish scientists: 1) Spanish scientists working abroad (SSA), 2) scientists who have returned to Spain, after practicing science for at least one year abroad (SRS), and 3) young researchers in Spain (YRS). The latter group is added because these researchers are highly susceptible to going abroad in order to obtain cumulative advantages [22, 25] and especially new opportunities to develop their scientific career [26, 27], but have not yet initiated their move abroad. Said group has been defined as scientists that have begun Doctorate programmes in Spain and have continued to work in science up to the age of 41. This study was carried out by means of an on-line survey between December 2016 and April 2017.

### Ethics statement

The *Ethics Committee of the Madrid Open University* (MOU) has approved this study. All participants gave their informed consent for participating in the study, as embedded in the questionnaire. Completing the survey was voluntary and anonymous. The authors of this study have not interacted with the participants. The data from this study is shown in the tables and in the supporting information. Researchers wishing to access any kind of information from this study can contact the authors, as long as the anonymity of the participants is respected.

### Survey and data analysis

The report from Global Entrepreneurship Monitor on the entrepreneurship phenomena, GEM, is of great value because it provides empirical data on entrepreneurship which can be compared internationally and over time [48]. Therefore, in order to guarantee the validity of the survey, the following definitions established in the GEM Adult Population Survey have been used, meaning that such comparisons can be made with international [49] and national [41] data. Also, in order to discover the fundamental reasons for the effectivity of scientific mobility for the promotion of entrepreneurship and intrapreneurship, questions related in this regard to previous literature were included in the survey [30–32]. Added to these reasons is specific training in entrepreneurship, as an important source of human capital that an entrepreneur [50] has and that may have been acquired by researchers. With this knowledge base, fourteen variables were studied for the three selected groups in turn. These variables have been grouped into the following three blocks:

* Block 1 (*type of entrepreneurship*).—In this block, the researchers have to identify their (1) *type of entrepreneurship* according to the activities that they have been most involved in: entrepreneurship in the past 3 and a half years, intrapreneurship in the past three years, and none of the above activities. The definitions of entrepreneurship and intrapreneurship are the following:

- Entrepreneurship.—Is a process that starts with an idea, continues with actions to put it into practice, is launched onto the market, enters a consolidation phase and then moves on to the consolidated phase when it survives for more than 3 and a half years. Another possible outcome is that the promotor or promotors leaves the project, either to pass the initiative to other owners or to close it completely.
- Intrapreneurship.—Is a process carried out by a person who is involved in the leadership and development of an entrepreneurial initiative for the organisation where they have worked for the past 3 years (University, Public Research Institutions, Companies, and others). Some examples are the creation of a new product/service, a new company or a new business unit, among others.

* Block 2 (*stage of entrepreneurship and effectivity of scientific experience for entrepreneurship*).- Only those researchers who have identified themselves as entrepreneurs for the past 3 years in the previous section have to select the (2) *stage of entrepreneurship*. In order to classify these stages, the following definitions for entrepreneurship and its various stages have been taken into account:

- (a) Potential entrepreneurship.- Entrepreneurship stage of a person who intends to start a new business in the next 3 years.
- (b) Nascent entrepreneurship.- Entrepreneurship stage of a person who is starting a new business in which they have invested time and effort in order to create it, but who has not paid salaries for more than 3 months.
- (c) New entrepreneurship.- Entrepreneurship stage of a person who has a business that has paid salaries for more than 3 months but not more than 42 months and that, therefore, has not been consolidated. Total Early-stage Entrepreneurial Activity, TEA, is an indicator that groups together the percentages of entrepreneurs by the two previous stages: nascent and new.
- (d) Established company.- Stage of a person who has a business that is well established in the market after having paid salaries for more than 42 months.
- (e) Discontinuation: transfer and closure: Stage in which the venture has been passed on to other people or closed completely in the last 12 months.

Also in this block are a series of variables taken from the literature mentioned in the introduction to the paper [30–32, 50] for which the researcher’s experience may have been effective: (3) *Recognition of business opportunities for entrepreneurship*, (4) *Acquisition of entrepreneurship basic knowledge (applying for public or private funding*, *drawing up a business plan*, *marketing*, *human resources*, *organization*, *among others) for the project(s)*, (5) *Access to networks of high entrepreneurial culture to support the project(s)*, (6) *Access to financial resources to support the project(s)*, (7) *Development of entrepreneurial skills (leadership*, *problem solving*, *organization*, *planning*, *decision making*, *among others) for the project(s)* and (8) *Influence from academic entrepreneurs/intrapreneurs*.

* Block 3 (*effectivity of the scientific experience for intrapreneurship*).- The same variables established for the above researchers, who have carried out entrepreneurial activities in the past three years, have been adapted for this kind of entrepreneurship which is intrapreneurship. These variables have been compiled only for those researchers who have carried out intrapreneurial activities. These variables, upon which the researcher’s experience may have had an effect, are the following: (9) *Recognition of business opportunities for entrepreneurship*, (10) *Acquisition of entrepreneurship basic knowledge (applying for public or private funding*, *drawing up a business plan*, *marketing*, *human resources*, *organization*, *among others) for the project(s)*, (11) *Access to networks of high entrepreneurial culture to support the project(s)*, (12) *Access to financial resources to support the project(s)*, (13) *Development of entrepreneurial skills (leadership*, *problem solving*, *organization*, *planning*, *decision making*, *among others) for the project(s)* and (14) *Influence from academic entrepreneurs/intrapreneurs*.

Regarding the variables relating to the effectivity of the experience, with those in the SSA and SRS groups, these variables made reference to the degree to which the experience acquired in their scientific mobility abroad has been effective for their entrepreneurship or intrapreneurship. The YRS have made their career in Spain, so these variables made reference to the degree to which the experience acquired in their scientific career has been effective for their entrepreneurship or intrapreneurship.

A Likert scale from 0 to 10 points was used to assess these variables, in which higher values mean a greater effectivity and lower values a lower effectivity. As can be seen from the results, only the percentages between the valuation “highly effective” (6) or “extremely effective” (10) have been taken into account.

Also, in order to support the implementation of effective mobility policies that stimulate entrepreneurship and intrapreneurship, the contributions of various authors have been taken into account in the study of the general profile of the participants. From the work of Franzoni, Scellato and Stephan [21], a new question was adapted about the possibility of returning in the future with various options: “yes”, “no”, “depends on the job opportunities”, or “perhaps part-time or at the end of career”. In our study, this question is formulated in exactly the same way for the SSA. For the SRS, this question is asked regarding going abroad again. For the YRS, we tried to ascertain the likelihood of these researchers going abroad to continue with their research career, given that this group has not yet moved and the importance of their research career to the YRS [26, 27]. To complete this profile, we followed the approach of the study by Baruffaldi and Landoni [20] in which it was concluded that the probability of researchers returning increases with a more temporary professional situation and with reasons for moving that are not related to improving job opportunities. According with this study, the following variables were taken into account: knowledge area, gender, position in the organization and geographic location.

### Survey development and properties

In this work the following steps were followed in order to ensure the quality of the study: (1) selection and definition of the variables in the study (2) choice of the medium used to obtain the answers from the participants, (3) description of the instructions for the participants, and (4) development of a pilot test of the rough draft of the survey.

This pilot test was carried out on a group of ten researchers from different areas that were informed of the objectives and the variables of the study. Their answers were used to address the clarity of the questions and the variables and the need to incorporate or eliminate certain variables in order to improve the results. With the information from this pilot test, the only change was to the definition of intrapreneurship including a description of the organization in which the scientists might work (Universities, Public Research Institutions, Companies, and others), in order to clarify this point.

### Participants

As has been shown previously, various studies [20, 21] demonstrate that it is difficult to follow up with researchers abroad. In the case of SSA various attempts have been made to carry out a census, with results between approximately 1000 and 2000 researchers [44, 51–53], but there is no official data on the number of persons making up this group. Likewise, as regards the SRS, there are no official figures, although 716 Spanish scientists were attracted back by the *Ramón y Cajal* programme in 2008, according to the *Ministerio de Ciencia e Innovación* [54]. Nor are there any official figures for the number of YRS.

Therefore, there are no available data for the population and profile of these researchers. In order to overcome this problem, the procedure of Baruffaldi and Landoni [20] has been followed who, as has previously been shown, had similar difficulties in a study on researchers abroad. To this end, and in harmony with the objectives of the study, the data from the participants has been compiled by associations and entities interested in supporting the development of the best conditions for scientific careers in Spain. For the SSA, the data has been compiled from lists of the following associations: *Society of Spanish Researchers in the United Kingdom /Comunidad de Científicos Españoles en el Reino Unido* (SRUK/CERU), *Científicos Españoles en la República Federal de Alemania* (CERFA), *Asociación de Científicos Españoles en Japón* (ACE Japón), *Españoles Científicos en Estados Unidos* (ECUSA), *Asociación de Científicos Españoles en Suecia/Association of Spanish Scientists in Sweden* (ACES/ FSFS), *Spanish Research in Australia-Pacific/Investigadores Españoles en Australia-Pacífico* (SRAP/IEAP), *Científicos Españoles en Dinamarca/Spanske Forskere i Danmark* (CED), *Asociación de Investigadores Españoles en la República Italiana* (ASIERI), *Red de Científicos Españoles en México* (RECEMEX) and *Asociación de Investigadores Españoles en Irlanda* (SRSI). The data for SRS has been compiled from the association *Científicos Retornados a España* (CRE) and from *Fundación Universidad-Empresa* (FUE), which also supplied the data for young researchers in conjunction with *Federación de Jóvenes Investigadores* (FJI), *Colegio Oficial de Físicos* (COFIS), *Federación Española de Biotecnólogos* (FEBiotec), *ARATECH* y *Centro de Innovación de la Universidad de Oviedo*.

Combining all of the data from these associations gives a total of 4,668 individuals for a final sample of 364 subjects. In this case, a maximum error sample of ^±^2.68% is assumed (confidence level 95%), which is within the parameters required for this kind of sample [55]. Also, the percentage of responses obtained are rather lower than in the Baruffaldi and Landoni [20] study (18%), but much higher (7.80%) than those gathered for the GEM Spain Report 2015 [41] (0.083%; confidence level: 95.5% sample error: ^±^0.62%), in a country such as Spain with limited activity in scientific companies and, possibly because of this, not much of a business culture within this group [42, 43].

From 19 December 2016 to 30 April 2017 the associations and entities distributed the survey to their scientists via email. Approximately every fortnight the researchers carrying out the study informed the associations and entities about the number of responses obtained. With this information, these organizations continued to send out the survey via email. At the end of April, the last call was sent out via email. The survey was closed on 30 April 2017.

## Results

### Profile of the participants

**Fig 1:**
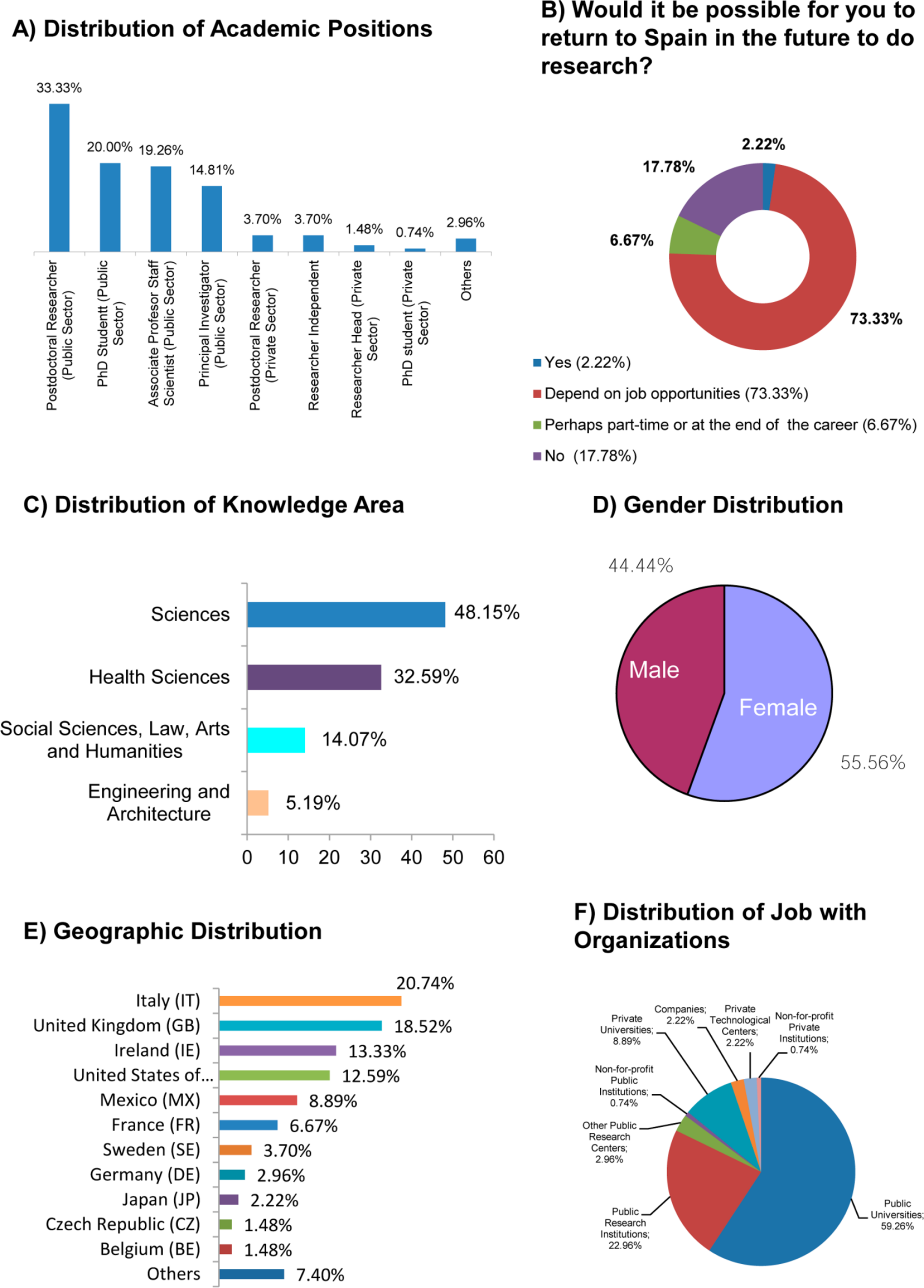
Profile of Spanish scientists abroad (n = 135). (A) Distribution of academic positions in percentage. (B) Distribution of the opinion about a hypothetical return to Spain in percentages. (C) Distribution of knowledge area in percentage. (D) Gender distribution in percentage. (E) Distribution of geographic locations in the top-11 countries. Others refers to "Argentina (0.74%), Chile (0.74%), China (0.74%), Dominican Republic (0.74%), Ecuador (0.74%), Netherlands (0.74%), Norway (0.74%), Portugal (0.74%), Switzerland (0.74%), Venezuela (0.74%)". (F) Distribution of jobs with organizations within the Public and Private Sectors.

A significant majority of SSA hold temporary positions within their organization, since only is of 35.55% (48/135, Fig 1A) the total of Associate Professors o Scientific Staff at the Public Sector (19.26%, 26/135), Principal Investigators (14.81%, 20/135) and Researchers Head of unit in the private sector (1.48%, 2/135). Also, a majority of SSA, 73.33%, (99/135, Fig 1B) could return to Spain depending on job opportunities. The Sciences and Health Sciences are the knowledge areas that dominate among SSA, with over 80% (109/135) of researchers working in these fields (Fig 1C). Regarding gender, women make up the majority of this group with 55.56% (75/135, Fig 1D). Italy (20.74%, 28/135), the United Kingdom (18.52%, 25/135), Ireland (13.33%, 18/135) and the United States (12.59%, 17/135) are the countries that have the greatest number of SSA followed quite a long way behind by Mexico (8.89%, 12/135), which leads the group of the other countries (Fig 1E). Public Universities and Public Research Institutions are the institutions most represented by SSA with over 82% (111/135) (Fig 1F).

**Fig 2:**
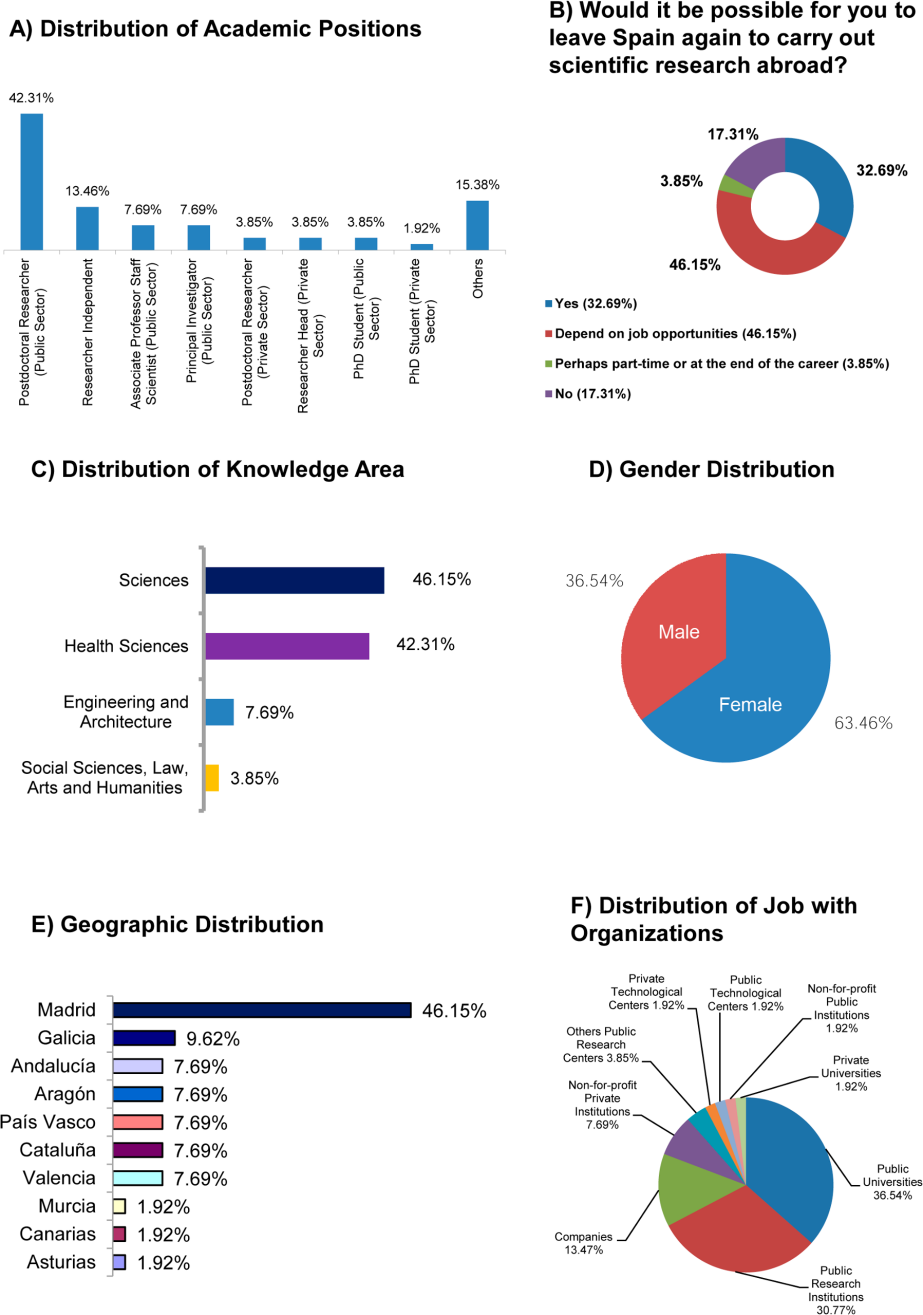
Profile of scientists returned to Spain (n = 52). (A) Distribution of academic positions in percentage. (B) Distribution of opinion about a hypothetical leaving Spain again in percentage. (C) Distribution of knowledge area in percentage. (D) Gender distribution in percentage. (E) Distribution of geographic locations in the autonomous regions. (F) Distribution of job with organizations within the Public and Private Sectors.

As regards SRS, only 19.23% (10/52) have a permanent position in their organization (7.69%, 4/52, Associate Professors o Scientific Staff at the Public Sector, 7.69%, 4/52, Principal Investigators and Researchers Head of unit in the private sector 3.85%, 2/52, Fig 2A). Also, a significant percentage, 46.15% (24/52, Fig 2B), of researchers in this group would leave again depending on job opportunities. Likewise, a significant majority of over 88% (46/135, Fig 2C) belong to knowledge areas in the Sciences and Health Sciences. By gender, women make up the majority of this group with 63.46% (33/52, Fig 2D). According to Fig 2E, almost half of SRS carry out their activities in the Community of Madrid (46.15%, 24/52), with other significant percentages relating to the following communities: Galicia (9.62%, 5/52), Andalucía (7.69%, 4/52), Aragón (7.69%, 4/52), País Vasco (7.69%, 4/52), Cataluña (7.69%, 4/52) and Valencia (7.69%, 4/52). The main types of organization in this group are Public Universities and Public Research Institutions with over 67% (35/52, Fig 2F).

**Fig 3:**
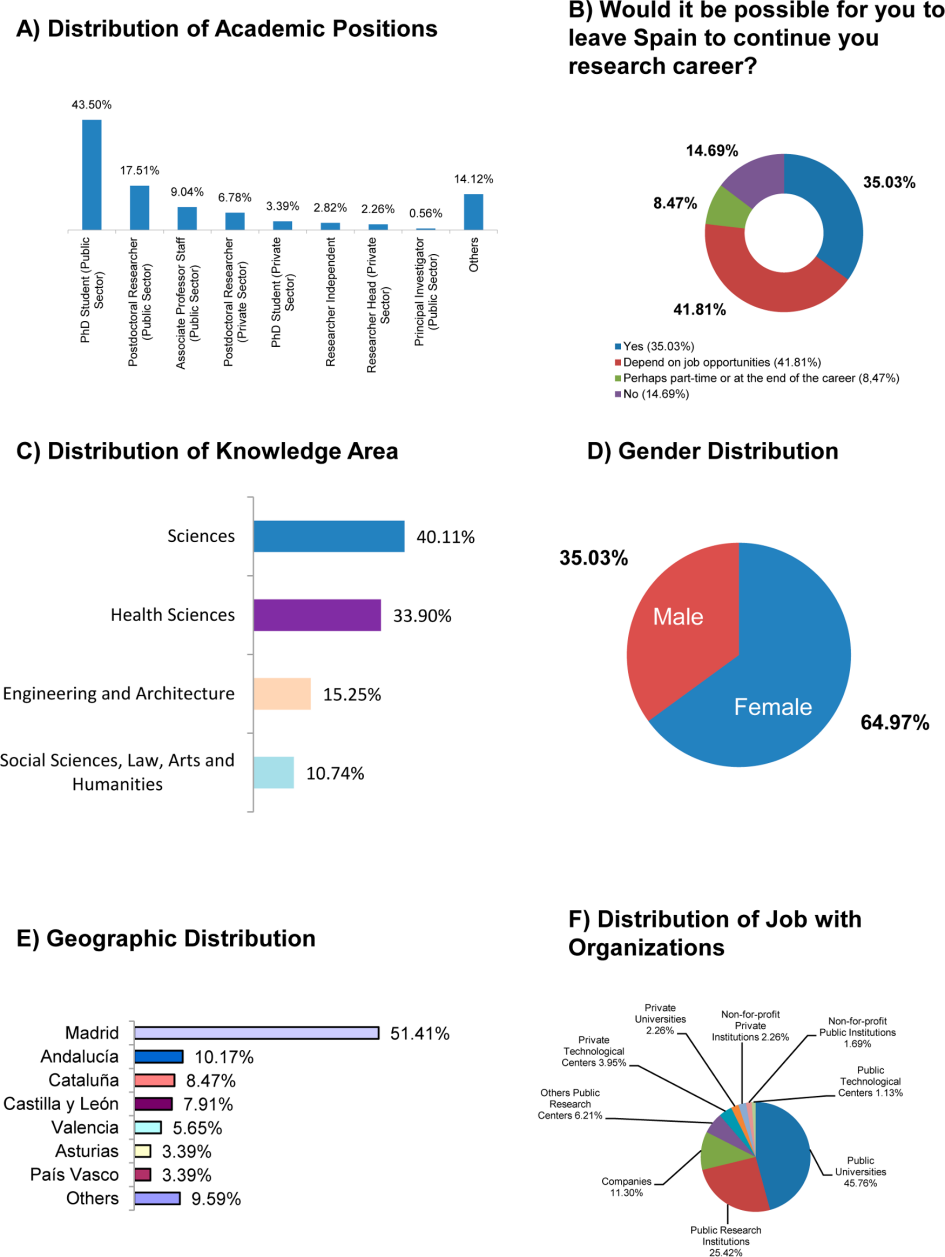
Profile of young researchers in Spain (n = 177). (A) Distribution of academic positions in percentage. (B) Distribution of the opinion about a hypothetical departure from Spain in percentages. (C) Distribution of knowledge area in percentage. (D) Gender distribution in percentage. (E) Distribution of geographic locations in the top-7 indicating autonomous regions. Others refers to "Región de Murcia (1.69%), Aragón (1.13%), Cantabria (1.13%), Castilla-La Mancha (1.13%), Extremadura (1.13%), Galicia (1.13%), Navarra (1.13%), Canarias (0.56%), La Rioja (0.56%)". (F) Distribution of jobs with organizations within the Public and Private Sectors.

On the other hand, only 11.86% (21/177, Fig 3A) of YRS have a permanent position (9.04%, 16/177, Associate Professors o Scientific Staff at the Public Sector, 0.56%, 1/177, Principal Investigators and Researchers Head of unit in the private sector, 2.26%, 4/177). Likewise, a significant percentage, 41.81% (74/177, Fig 3B), of researchers in this group would leave Spain in order to continue working in science depending on job opportunities. Also, for YRS who took part in the study, Sciences and Health Sciences are the best-represented knowledge areas, between them over 74% (131/177, Fig 3C). As regards gender distribution, women make up the majority of this group with 64.97% (115/177, Fig 3D). As was the case with SRS, a large majority of YRS (51.41%, 91/177, Fig 3E) belong to the Community of Madrid, with other communities some way behind with lower percentages: Andalucía (10.17%, 18/177), Cataluña (8.47%, 15/177), Castilla y León (7.91%, 14/177) and Valencia (5.65%, 10/177). As with the previous cases, the main types of organization represented in this group are Public Universities and Public Research Institutions with over 71% (126/177, Fig 3F).

### Type of entrepreneurship

As can be seen from Table 1, the group most involved in entrepreneurial activities is that of the SSA with 32.59% (44 out of 135: 9.63% -13/135- in entrepreneurial activities and 22.96% -31/135- in intrapreneurial activities). The next group most involved in this kind of activity is that of the SRS with 26.92% (14 out of 52: 11.54% -6/52- in entrepreneurial activities and 15.38% -8/52- in intrapreneurial activities). The YRS group is in last place with 19.77% of researchers involved in these kinds of activities (35 out of 177: 5.65% in entrepreneurial activities -10/177- and 14.12% -25/177- in intrapreneurial activities). The intrapreneurial activities stand out among these entrepreneurial activities, with rates much higher than those for entrepreneurship, especially for SSA, with the highest rate of intrapreneurship (22.96%, 31/135) which is over 13 percentage points higher than their entrepreneurship rate (9.63%, 13/135). This significant rate of intrapreneurship by SSA is followed by the SRS (15.38%, 8/52), and the YRS are not far behind with (14.12%, 25/177%). These data on intrapreneurship are particularly important for a country like Spain, which has an intrapreneurship rate of 1.1%, according to data from reports from GEM Global Report 2015/2016 [49] and GEM Spain Report 2015 [41], placing it in 22nd place out of 24 economies in its group of innovation-based economies. In fact, the rates obtained are high when compared with the average of these countries in the group of innovation-based economies (5.1%) and the country with the highest rate, which is Norway with 9.9%.

**Table 1:** Type of entrepreneurship for each group of researchers.

Type of entrepreneurship	Groups of researchers (%)^a^		
	SSA^b^	SRS^c^	YRS^d^
**Entrepreneurship in the last 3 and a half years**^**e**^	9.63%	11.54%	5.65%
**Intrapreneurship in the last 3 years**^**f**^	22.96%	15.38%	14.12%
**None of the above activities**	67.41%	73.08%	80.23%

^a^Percentage of researchers “involved in” or “not involved in” entrepreneurial activities.

^b^Spanish scientists abroad.

^c^Scientists returned to Spain.

^d^Young researchers in Spain.

^e^Entrepreneurship [41, 49].- Is a process that starts with an idea, continues with actions to put it into practice, is launched onto the market, enters a consolidation phase and then moves on to the consolidated phase when it survives for more than 3 and a half years. Another possible outcome is that the promotor or promotors leaves the project, either in order to pass the initiative on to other owners or to close it completely.

^f^Intrapreneurship [41, 49].- Is a process carried out by a person who is involved in the leadership and development of an entrepreneurial initiative for the organization where they have worked for the past 3 years (University, Public Research Institutions, Companies, and others). Some examples are the creation of a new product/service, a new company or a new business unit, among others.

### Stage of entrepreneurship and effectivity of scientific experience for entrepreneurship

According to Table 2, the group with the highest percentage of potential entrepreneurs is the SSA, with 5.19% (7/135), followed by the SRS with 3.85% (2/52) and lastly the YRS with 2.26% (4/177). Taking into account international [49] and national [41] data, these rates of groups of researchers are lower than the rate for Spain (6.1%, 24th place), the average for innovation-based economies (14.2%), and Taiwan (27.5%), which is the leader in adults involved in these stage of entrepreneurship.

**Table 2:** Stages of the entrepreneurship process.

	Groups of researchers (%)^a^			GEM data on innovation-based economies (%) [41, 49]		
	SSA^b^	SRS^c^	YRS^d^	Spain (position)^e^	Average	Leading country
**Potential entrepreneurship**^**f**^	5.19%	3.85%	2.26%	6.1% (24)	14.2%	27.5% (Taiwan)
**Nascent entrepreneurship**^**g**^	3.70%	1.92%	0.56%	2.1% (24)	5.3%	9.7% (Canada)
**New entrepreneurship**^**h**^	0.00%	1.92%	0.56%	3.6% (9)	3.4%	5.8% (Australia)
**Total Early-stage Entrepreneurial Activity, TEA**^**i**^	3.70%	3.84%	1.12%	5.7% (21)	8.5%	14.7% (Canada)
**Established company**^**j**^	0.00%	0.00%	0.00%	7.7% (9)	6.8%	13.1% (Greece)
**Discontinuation**^**k**^	0.74%	3.85%	2.26%	1.6% (21)	2.8%	0.9% (Puerto Rico)

^a^Percentage of researchers who have been entrepreneurs in the past three years identified by the stage of their entrepreneurship.

^b^Spanish scientists abroad.

^c^Scientists returned to Spain.

^d^Young researchers in Spain.

^e^Spain’s position out of the 24 innovation-based economies [41, 49].

^f^Potential entrepreneurship [41, 49].- entrepreneurship stage of a person who intends to start a new business in the next 3 years.

^g^Nascent entrepreneurship [41, 49].- entrepreneurship stage of a person who is starting a new business in which they have invested time and effort in order to create it, but who has not paid salaries for more than 3 months.

^h^New entrepreneurship [41, 49].- entrepreneurship stage of a person who has a business that has paid salaries for more than 3 months but not more than 42 months and that, therefore, has not been consolidated.

^i^Total Early-stage Entrepreneurial Activity, TEA [41, 49].- is an indicator that groups together the percentages of entrepreneurs by the two previous stages: nascent and new.

^j^Established company [41, 49].- stage of a person who has a business that is well established in the market after having paid salaries for more than 42 months.

^k^Discontinuation: transfer and closure [41, 49].- stage in which the venture has been passed on to other people or closed completely in the last 12 months.

Regarding nascent entrepreneurship, SSA is also the leading group for this stage of entrepreneurship with 3.70% (5/135). This is followed by the SRS with a rate of 1.92% (1/52), and again the YRS are in last place in this area with 0.56% (1/177). All of these percentages are lower than the average for innovation-based economies (5.3%) and lower than the country with the highest rate in this area (Canada with 9.7%) but, for the group of SSA only (3.70%), it is higher than the rate for Spain (2.1% in 24th place).

As regards new entrepreneurship, the rates are the same as the above areas for SRS (1.92%, 1/52) and for YRS (0.56%, 1/177) and are reduced to zero for the SSA. Given this data, all of the rates are below the Spanish average (3.6%, 9th place), the average rate for innovation-based economies (3.4%) and the rate of the leading country in this area (Australia with 5.8%).

Given the percentages for the latter two stages, the SRS have the highest TEA (3.84%, 2/52), followed by the SSA (3.70%, 5/135) and the YRS are again in last place (1.12%, 2/177). As with the previous area, all of the percentages are lower than the average for Spain (5.7%, 21st place), the average for innovation-based economies (8.5%) and the percentage for the leading country in this stage of entrepreneurship (Canada with 14.7%).

As regards the stage of established companies, both the national rate (7.7%, 9th place) and international rates (6.8% average for innovation-based economies and 13.1% for Greece as the leading country) are higher, since no researchers have reached this stage in their entrepreneurship.

The group with fewest researchers leaving their entrepreneurial activities is that of SSA with (0.74%, 1/135), followed by the YRS (2.26%, 4/177) and SRS (3.85%, 2/52) with the highest rate of discontinuation out of these groups. This rate for the SRS is above the Spanish average (1.6%, 21st place), the average rate for innovation-based economies (2.8%) and the rate of the leading country in this area (Puerto Rico with 0.9%). Likewise, the rate for YRS is between the rate for Spain and the average rate. Lastly, the rate for SSA is even higher than that of Puerto Rico, which is the leading country in this stage.

Regarding the effectivity of scientific experience for the entrepreneurship of the groups of researchers, as can be seen in Table 3, the effect on SSA with their scientific mobility abroad is noteworthy since over 60% of researchers in this group, in all of the variables, view this mobility as highly or extremely effective for their entrepreneurship. Particularly noteworthy were the variables *acquisition of entrepreneurship basic knowledge* with 84.62% (11/13) and *development of entrepreneurial skills*, also with 84.62% (11/13). The next group with a large number of variables that reach at least fifty percent that view this mobility as highly or extremely effective for their entrepreneurship is that of the SRS, although this group also has the lowest percentages of all of the groups with 33.33% (2/6) for *acquisition of entrepreneurship basic knowledge* and *development of entrepreneurial skills*. Finally, the YRS group with the experience gained in their scientific career in Spain only exceed the previous percentage of 50.00% for the variables *acquisition of entrepreneurship basic knowledge* with 60.00% (6/10) and *access to networks of high entrepreneurial culture* with 70.00% (7/10).

**Table 3:** Effectivity of scientific experience for the entrepreneurship of each group of researchers.

Variables	Group of researchers (%)^a^		
	SSA^b^	SRS^c^	YRS^d^
**Recognition of business opportunities for entrepreneurship**	61.54%	50.00%	40.00%
**Acquisition of entrepreneurship basic knowledge (applying for public or private funding, drawing up a business plan, marketing, human resources, organization, among others) for the project(s)**	84.62%	33.33%	60.00%
**Access to networks of high entrepreneurial culture to support the project(s)**	61.54%	50.00%	70.00%
**Access to financial resources to support the project(s)**	61.54%	50.00%	40.00%
**Development of entrepreneurial skills (leadership, problem solving, organization, planning, decision making, among others) for the project(s)**	84.62%	33.33%	40.00%
**Influence from academic entrepreneurs/intrapreneurs**	69.23%	66.67%	40.00%

^a^Percentage of researchers indicating “highly effective” or “extremely effective”.

^b^Spanish scientists abroad.

^c^Scientists returned to Spain.

^d^Young researchers in Spain.

### Effectivity of scientific experience for intrapreneurship

Again it is the two mobile scientific groups that present a greater effectivity of scientific experience gained abroad for their intrapreneurship, since, as can be seen from Table 4, all of the variables for these groups exceed fifty percent that consider that this scientific mobility has been highly or extremely effective for their intrapreneurship. The SSA are noteworthy with over 75% for variables such as *development of entrepreneurial skills* (87.10%, 27/31), *acquisition of entrepreneurship basic knowledge* (80.65%, 25/31) and *recognition of business opportunities for entrepreneurship* (77.42%, 24/31). Likewise, the SRS group also reached significant percentages of 75.00% (6/8) for variables such as *acquisition of entrepreneurship basic knowledge* and *development of entrepreneurial skills*. The YRS group is the only one with a variable such as *access to financial resources* (44.00%, 11/25) under the 50.00% threshold, although it does reach a significant percentage of over 75.00% for *development of entrepreneurial skills* (84.00%, 21/25).

**Table 4:** Effectivity of scientific experience for the intrapreneurship of each group of researchers.

Variables	Group of researchers (%)^a^		
	SSA^b^	SRS^c^	YRS^d^
**Recognition of business opportunities for entrepreneurship**	77.42%	62.50%	56.00%
**Acquisition of entrepreneurship basic knowledge (applying for public or private funding, drawing up a business plan, marketing, human resources, organization, among others) for the project(s)**	80.65%	75.00%	64.00%
**Access to networks of high entrepreneurial culture to support the project(s)**	51.61%	62.50%	56.00%
**Access to financial resources to support the project(s)**	61.29%	50.00%	44.00%
**Development of entrepreneurial skills (leadership, problem solving, organization, planning, decision making, among others) for the project(s)**	87.10%	75.00%	84.00%
**Influence from academic entrepreneurs/intrapreneurs**	61.29%	62.50%	52.00%

^a^Percentage of researchers indicating “highly effective” or “extremely effective”.

^b^Spanish scientists abroad.

^c^Scientists returned to Spain.

^d^Young researchers in Spain.

## Discussion

The results show that only a small percentage of SSA have a permanent position and the proportion of researchers that could return to Spain depending on job opportunities is very high. As regards the SRS, also few of them have a permanent position and a significant proportion would go abroad again depending on job opportunities. A significant percentage of YRS would also leave Spain in order to continue working in science depending on job opportunities and only a small proportion of researchers in this group have a permanent position.

According to these results and taking into account previous studies [21], SECTI agents can have an influence on achieving sufficient scientific mobility by generating the conditions to create these job opportunities or by directly creating such opportunities. Moreover, the small percentages of these three groups with permanent positions [20], confirm that SECTI agents can exercise this influence on the return and retention of the three groups.

The data shows that few members of the groups of Spanish scientists involved in scientific mobility view entrepreneurship as a job opportunity. Almost all of the rates obtained in the various stages of the entrepreneurship process defined by GEM [41, 49] are a lot lower than national and international rates, which is congruous with the small number of companies created by scientists, as shown in previous studies [32, 39]. However, the mobile groups of SSA and SRS separately show better figures than the YRS in these stages, with the exception of SSA in new entrepreneurship and discontinuation among SRS.

In terms of percentages, with the exception of the SRS group in *acquisition of entrepreneurship basic knowledge* and *development of entrepreneurial skills*, more mobile scientists gained effective experience for their entrepreneurship through their scientific mobility abroad. It is possible that those SRS have not significantly acquired this knowledge and these skills, since they planned in advance to divest the business to other persons or institutions in order to continue to concentrate on developing other ideas, which is reflected in the high discontinuation rate in this group. In view of this, it can be said that scientific mobility abroad promotes entrepreneurship, which is congruous with what has been set out in previous studies [29–32].

As regards intrapreneurship, this activity can be viewed as a job opportunity for these groups of scientists, since the rates reached are far higher than national or international levels. As above, the mobile groups of SSA and SRS separately show better figures than the YRS in this activity, but the figures obtained by the latter group are also significant. This data could be very important in order for a country such as Spain to move up the world rankings for intrapreneurship within innovation-based economies [41, 49] and also in order to be able to create more jobs and to be more competitive, as previously set out by the WEF [36].

Likewise, a greater percentage of mobile scientists acquired more effective experience for their intrapreneurship in their scientific mobility abroad, and although YRS also show adequate figures, except in *access to financial resources*, the general case of SSA is outstanding, especially for variables such as *development of entrepreneurial skills*, *acquisition of entrepreneurship basic knowledge*, and *recognition of business opportunities for entrepreneurship*. As was the case with entrepreneurship above, it can be said that scientific mobility abroad promotes intrapreneurship, which is also congruous with what has been set out in previous studies [29–32].

Given such positive results for scientific mobility abroad promoting entrepreneurship and intrapreneurship, it is appropriate that the conditions and job opportunities be created in order to develop all of this entrepreneurial and intrapreneurial potential. In fact, the main recommendations for entrepreneurial activity from the experts set out in the GEM Spain Report 2015 [41] are congruous with these results.

Firstly, those experts highlight financial support. In this regard, both for entrepreneurship and intrapreneurship, the mobile groups present greater percentages of *access to financial resources to support the project(s)*, so supporting scientific mobility abroad could enable the Spanish economy to receive more economic funds for its entrepreneurial activities.

Education and training is the next aspect highlighted by the experts. As regards entrepreneurship, the SSA is the group with the greatest percentages both for *acquisition of entrepreneurship basic knowledge* and *development of entrepreneurial skills* and although the SRS is the group with the lowest percentages in these variables, the sum of these percentages of two mobile groups is higher than that of the YRS. For intrapreneurship, the percentages are higher for all of the groups, with the SSA group being especially outstanding. In view of this, again supporting scientific mobility abroad can enable the Spanish economy to achieve greater experience and training in order to carry out entrepreneurial activities.

Lastly, as a main recommendation the experts highlight government policies, some of which could be steered according to the results obtained in this work. As regards SSA, new companies and their associations could forge closer collaboration with them, since this is not out of reach [47], incorporating the researchers’ innovative initiatives and ensuring that these can be transferred and strengthened within the country [56]. These researchers know how other countries think, they have professional contacts, and they contribute significant competencies such as linguistic skills, which means that they could be used as a link between scientific organizations and foreign companies. In exchange, these new companies and their associations could provide SSA with the professional and economic recognition necessary in order to create favourable conditions for their return.

For the SRS, among the measures to facilitate their return the main ones are stable funding, salary or recognition for their scientific career, but a high percentage also point to the Social Responsibility of the organization as a measure for facilitating their return [45]. Likewise, a series of social capabilities that can be offered such as good working conditions and employee benefits which are decisive factors for researchers returning from abroad [46]. These complementary social advantages can be intensified by means of public-private partnerships, obtaining the necessary resources from the new companies and their associations (for example, employing the researcher’s spouse or paying for childcare). This will reduce the gap between the competitive advantages that other countries offer researchers and will give them greater flexibility in order to begin these new projects with companies or to implement new lines of research. These social measures can also be used to get researchers into the workforce of new companies and their associations, which would be another job opportunity for SRS.

The information obtained in this work has implications for improving entrepreneurship and intrapreneurship, since the knowledge acquired with good scientific mobility means that opportunities can be recognized, gives access to networks of high entrepreneurial culture and to financial resources, the acquisition of entrepreneurship basic knowledge, the development of entrepreneurial skills and the influence from academic entrepreneurs and intrapreneurs. This knowledge obtained abroad can be used in the creation of new technology companies or in the implementation of projects of this kind in existing organizations or companies with the potential to make them into leaders in innovation and generating employment.

### Limitations and future lines of investigation

The data obtained to support these conclusions are trustworthy and consistent with other studies, but they have the limitation of coming from a small sample of Spanish researchers. In order to ratify the validity of these conclusions in future studies this number will be increased.

Also, it has been necessary to use data from various associations and institutions, since there is no data on the total members of the participating groups. For this reason, the results obtained cannot be applied generally to all scientists in these groups. Likewise, this study evaluates the role and effective reasons for scientific mobility for the promotion of entrepreneurship and intrapreneurship, but not its evolution, so in future studies the time horizon will be extended by means of a longitudinal design. Similarly, the results of this study promote the continued study into the potential of collaboration between the groups involved in scientific mobility and new companies and their associations in order to promote entrepreneurship and to determine whether this can generate the right conditions for the return and retention of researchers to their home countries.

## Conclusions

Taking into account all that has been set out previously, this study has provided data which shows that groups of mobile scientists are more involved in entrepreneurial activities and, especially in intrapreneurial activities. Experience acquired abroad by these groups has also been more effective for promoting these activities and, as above, especially for intrapreneurs. All of this shows that scientific mobility promotes entrepreneurship and above all intrapreneurship. Therefore SECTI agents should intensify appropriate policies for scientific mobility in order to use this potential in order to benefit economic growth, creating jobs and the right balance between local and global science.

## Supporting information

S1 Questionnaire Questionnaire for Spanish researchers abroad-Cuestionario para los científicos españoles en el exterior. (DOC)

S2 Questionnaire Questionnaire for scientists returned to Spain-Cuestionario para los científicos retornados a España. (DOC)

S3 Questionnaire Questionnaire for young researchers in Spain-Cuestionario para los jóvenes investigadores en el España. (DOC)

S1 File Spanish scientists abroad-Científicos españoles en el exterior. (ODS)

S2 File Scientists returned to Spain-Científicos retornados a España. (ODS)

S3 File Young researchers in Spain-Jóvenes investigadores en España. (ODS)

## Abbrevations

ACE Japón: Asociación de Científicos Españoles en Japón
ACES: Association of Spanish Scientists in Sweden/Asociación de Científicos Españoles en Suecia
AES: Acción Estratégica de Salud
ASIERI: Asociación de Investigadores Españoles en la República Italiana
CED: Spanske Forskere i Danmark/Científicos Españoles en Dinamarca
CERFA: Científicos Españoles en la República Federal de Alemania
COFIS: Colegio Oficial de Físicos
ECUSA: Comunidad de Españoles Científicos en Estados Unidos
EEA: Employee Entrepreneurial Activity
FEBiotec: Federación Española de Biotecnólogos
FECYT: Spanish Foundation for Science and Technology /Fundación Española para la Ciencia y la Tecnología
FJI: Federación de Jóvenes Investigadores
FUE: Fundación Universidad-Empresa
GEM: Global Monitor Entrepreneurship
MEC: Ministerio de Educación y Ciencia
MOU: Madrid Open University
RECEMEX: Red de Científicos Españoles en México
R&D&i: Research, Development and Innovation
RedIEX: Sistema de Comunicación con Investigadores en el Exterior
SECTI: Sistema Español de Ciencia y Tecnología
SNS: Sistema Nacional de Salud
SRAP/IEAP: Spanish Researchers in Australia-Pacific/Investigadores Españoles en Australia-Pacífico
SRS / CRE: Scientists returned to Spain / Científicos retornados a España
SRSI: Asociación de Investigadores Españoles en Irlanda
SRUK/CERU: Society of Spanish Researchers in the United Kingdom/Comunidad de Científicos Españoles en el Reino Unido
SSA / CIEX: Spanish scientists abroad / Científicos españoles en el exterior
TEA: Total Early-stage Entrepreneurial Activity
WEF: World Economic Forum
YRS / JIES: Young researchers in Spain / Jóvenes investigadores en España
