# Supplementary material for: Entrepreneurship, intrapreneurship and scientific mobility: The Spanish case
Source: PLoS One. 2018 Sep 5;13(9):e0201893. doi: 10.1371/journal.pone.0201893 (PMC6124723; doi:10.1371/journal.pone.0201893)
Supplement: S3 Questionnaire — (DOC) [file pone.0201893.s003.doc]

**Cuestionario para los jóvenes investigadores en España (JIES)**

Questionnaire for Young researchers in Spain (YRS)

| **Indique el tipo de organización donde realiza su labor investigadora principal.**  Indicate the type of organization where you perform your main research work. | | |
| --- | --- | --- |
| **Opción**  Answer | **Cuenta**  Account | **Porcentaje**  Percentage |
| **Universidad pública**  Public University |  |  |
| **Universidad privada**  Private University |  |  |
| **Organismo público de investigación**  Public Research Institution |  |  |
| **Otros centros de I+D de las Administraciones Públicas**  Other Public Research Centers |  |  |
| **Centro tecnológico de propiedad y gestión pública**  Public Technological Center |  |  |
| **Centro tecnológico de propiedad y gestión privada**  Private Technological Center |  |  |
| **Entidad pública sin ánimo de lucro**  Non-for-profit Public Institution |  |  |
| **Entidad privada sin ánimo de lucro**  Non-for-profit Private Institution |  |  |
| **Gran empresa (igual o superior a 250 empleados)**  Large company (equal or more than 250 employees) |  |  |
| **Mediana empresa (menos de 250 empleados)**  Medium company (less than 250 employees) |  |  |
| **Pequeña empresa (menos de 50 empleados)**  Small company (less than 50 employees) |  |  |
| **Microempresa (menos de 10 empleados)**  Microcompany (less than 10 employees) |  |  |

| **Señale el área de conocimiento objeto de esta labor investigadora.**  Select the knowledge area ​for your research work. | | |
| --- | --- | --- |
| **Opción**  Answer | **Cuenta**  Account | **Porcentaje**  Percentage |
| **Ciencias**  Sciences |  |  |
| **Ciencias de la Salud**  Health Sciences |  |  |
| **Ciencias Sociales, Jurídicas, Artes y Humanidades**  Social Sciences, Law, Arts and Humanities |  |  |
| **Ingeniería y Arquitectura**  Engineering and Architecture |  |  |

| **Seleccione Comunidad o Ciudad Autónoma en caso de haber elegido España en la pregunta anterior**  Have you chosen Spain in the previous question, please select Region or Autonomous City as your answer below | | |
| --- | --- | --- |
| **Opción**  Answer | **Cuenta**  Account | **Porcentaje**  Percentage |

| **Identifique el grupo al que pertenece dentro del colectivo investigador:**  Identify your academic position | | |
| --- | --- | --- |
| **Opción**  Answer | **Cuenta**  Account | **Porcentaje**  Percentage |
| **Investigador predoctoral del sector público**  PhD Student at  the Public Sector |  |  |
| **Investigador postdoctoral del sector público**  **P**ostdoctoral Researcher at the Public Sector |  |  |
| **Investigador contratado doctor o científico de plantilla del sector público**  Associate Professor or Scientific Staff at the Public Sector |  |  |
| **Investigador titular (senior lecturer, reader, professor, team leader)**   Principal Investigator (Senior Lecturer, Reader, Professor, Team Leader) |  |  |
| **Investigador predoctoral del sector privado**  PhD Student at  the private sector |  |  |
| **Investigador postdoctoral del sector privado**  Postdoctoral researcher at the private sector |  |  |
| **Investigador Jefe de Equipo en el sector privado**  Researcher Head of unit in the private sector |  |  |
| **Investigador independiente**  Independent Researcher |  |  |
| **Otros**  Others |  |  |

| **Sexo:**  **Gender:** | | |
| --- | --- | --- |
| **Opción**  Answer | **Cuenta**  Account | **Porcentaje**  Percentage |
| **Femenino**  Female |  |  |
| **Masculino**  Male |  |  |

| **¿Es posible que Usted se marche al exterior para continuar efectuando ciencia?**  Would it be possible for you to leave Spain to continue your research career? | | |  |  |  |
| --- | --- | --- | --- | --- | --- |
| **Opción**  Answer | **Cuenta**  Account | **Porcentaje**  Percentage |  |  |  |
| **Si**  Yes |  |  | | | |
| **Depende de las oportunidades laborales**  Depends on the job opportunities |  |  | | | |
| **Quizás a tiempo parcial o al final de mi carrera**  Perhaps part-time or at the end of career |  |  | | | |
| **No**  No |  |  | | | |

**SCIENTIFIC ENTREPRENEURSHIP/INTRAPRENEURSHIP**

EMPRENDIMIENTO/INTRAEMPRENDIMIENTO CIENTÍFICO

**A continuación se realizarán una serie de preguntas relacionadas con el emprendimiento y el intraemprendimiento de su colectivo científico, para lo que se recomienda tener en cuenta los siguientes conceptos:**

In the following, we are giving you a series of questions related to entrepreneurship and intrapreneurship as they relate to your scientific collective. In order to answer them, it is recommended to take into account the following concepts:

**- Emprendimiento.- Es un proceso que se inicia con la generación de una idea, continua con las acciones de su puesta en marcha, se lanza al mercado, entra en una fase de consolidación y pasa a la fase consolidada cuando sobrevive más de 3 años y medio. Otro destino posible es el abandono del promotor o promotores, ya sea para traspasar la iniciativa a otros propietarios o para cerrarla de manera definitiva.**

- Entrepreneurship.- Is a process that starts with the generation of an idea, continues with the actions to put it into practice, it is then launched onto the market, enters a consolidation phase and then moves on to the consolidated phase when it survives for more than 3 and a half years. Another possible outcome is that the promotor or promotors leaves the project, either in order to pass the initiative on to other owners or to close it completely.

**- Intraemprendimiento.- Es un proceso realizado por una persona que se ha involucrado en el liderazgo y desarrollo de una iniciativa emprendedora para la organización en la que ha trabajado en los últimos 3 años (Universidad, Organismo Público de Investigación, Empresa, entre otras). Algunos ejemplos pueden ser la creación de un nuevo producto/servicio, una nueva empresa o nueva unidad de negocio, entre otros).**

- Intrapreneurship.- Is a process carried out by a person who is involved in the leadership and development of an entrepreneurial initiative for the organization where they have worked for the past 3 years (University, Public Research Institutions, Companies, and others). Some examples are the creation of a new product/service, a new company or a new business unit, among others.

| **De acuerdo con estas definiciones, ¿Usted ha realizado algunas de las siguientes actividades?. Seleccione solo aquella respuesta con la que se sienta más identificado.**  According to the definitions given above, have you ever performed any of the following activities? Please select only the answer that you feel most identified with. | | | |  |
| --- | --- | --- | --- | --- |
|  | **Emprendimiento en los últimos 3 años y medio**  Entrepreneurship in the last 3 and a half years |  |  |  |
|  | **Intraemprendimiento en los últimos 3 años**  Intrapreneurship in the last 3 years |  |  | |
|  | **Ninguna actividad de las anteriores**  None of the above activities |  |  | |

**- Solo para JIES emprendedores**

- Only for entrepreneurs YRS

| **¿En qué fase se encuentra su emprendimiento?**  In what phase is your entrepreneurship? | |  |  |
| --- | --- | --- | --- |
| A.1. | **Emprendimiento potencial.- Es el correspondiente a una persona con la intención de poner en mar­cha una nueva empresa en los próximos 3 años.**  Potential entrepreneurship - Is the one corresponding to a person with the intention of starting a new company in the next 3 years. | | |
| A.2. | **Emprendimiento naciente.- Es el correspondiente a una persona que está poniendo en marcha una empresa en la que se ha invertido tiempo y esfuerzo para su creación, pero que no ha paga­do salarios por más de 3 meses.**  Nascent entrepreneurship.- Is the one corresponding to a person who is starting a company in the creation of which he/she has invested time and effort, but has not paid salaries for more than 3 months. | | |
| A.3. | **Emprendimiento nuevo.- Es el correspondiente a una persona que posee un negocio que ha pagado salarios por más de 3 meses y no más de 42 meses, y que, por lo tanto, no se ha consolidado.**  New Entrepreneurship .- Is the one corresponding to a person who owns a business that has paid salaries for more than 3 months but not for more than 42 months, and therefore it is considered to be not consolidated. | | |
| A.4. | **Empresa consolidada.- Es el correspondiente a una per­sona que posee un negocio que ya se ha afianzado en el mercado tras haber pagado salarios por más de 42 meses.**  Established company.- This corresponds to a person who owns a business that is well established in the market after having paid salaries for more than 42 months. | | |
| A.5. | **Abandono: traspaso y cierre: El emprendimiento ha sido traspasado a otras personas o cerrado de manera definitiva en los últimos 12 meses.**  Discontinuation: transfer and closure: The venture has been passed to other people, or been definitively closed, in the last 12 months. | | |

| **Indique en qué medida la experiencia adquirida en su movilidad científica en el exterior ha sido efectiva para su emprendimiento en los siguientes aspectos:**  Please indicate to what extent your experience gained through your scientific mobility abroad has been effective for your entrepreneurship in the following aspects:  **Escala de Likert de 0 a 10: nada efectiva = 0; extremadamente efectiva = 10.**  Likert scale from 0 to 10: not effective at all = 0; extremely effective = 10. | | |
| --- | --- | --- |
| **Opción**  Answer | **Cuenta**  Account | **Porcentaje**  Percentage |
| **Reconocimiento de oportunidades de negocio para su emprendimiento**  Recognition of business opportunities for your entrepreneurship |  |  |
| **Adquisición de conocimientos básicos de emprendimiento (solicitud de financiación pública o privada, elaboración de un plan de negocios, comercialización, recursos humanos, organización, por ejemplo) para su/s proyecto/s**  Acquisition of entrepreneurship basic knowledge (applying for public or private funding, drawing up a business plan, marketing, human resources, organization, among others) for your/s project/s |  |  |
| **Acceso a redes de elevada cultura emprendedora para apoyar su/s proyecto/s**  Access to networks of high entrepreneurial culture to support your/s project/s |  |  |
| **Acceso a recursos financieros para apoyar su/s proyecto/s**  Access to financial resources to support your/s project/s |  |  |
| **Desarrollo de competencias emprendedoras (liderazgo, resolución de problemas, organización, planificación, toma de decisiones, entre otras) para su/s proyecto/s**  Development of entrepreneurial skills (leadership, problem solving, organization, planning, decision making, among others) for your/s project/s |  |  |
| **Influencia recibida por parte de emprendedores/intraemprendedores académicos**  Influence from academic entrepreneurs/intrapreneurs |  |  |

**- Solo para JIES intraemprendedores**

- Only for intrapreneurs YRS

| **Indique en qué medida la experiencia adquirida en su movilidad científica en el exterior ha sido efectiva para su intraemprendimiento en los siguientes aspectos:**  Please indicate to what extent your experience gained through your scientific mobility abroad has been effective for your intrapreneurship in the following aspects:  **Escala de Likert de 0 a 10: nada efectiva = 0; extremadamente efectiva = 10.**  Likert scale from 0 to 10: not effective at all = 0; extremely effective = 10.  . | | |
| --- | --- | --- |
| **Opción**  Answer | **Cuenta**  Account | **Porcentaje**  Percentage |
| **Reconocimiento de oportunidades de negocio para su emprendimiento**  Recognition of business opportunities for your entrepreneurship |  |  |
| **Adquisición de conocimientos básicos de emprendimiento (solicitud de financiación pública o privada, elaboración de un plan de negocios, comercialización, recursos humanos, organización, por ejemplo) para su/s proyecto/s**  Acquisition of entrepreneurship basic knowledge (applying for public or private funding, drawing up a business plan, marketing, human resources, organization, among others) for your/s project/s |  |  |
| **Acceso a redes de elevada cultura emprendedora para apoyar su/s proyecto/s**  Access to networks of high entrepreneurial culture to support your/s project/s |  |  |
| **Acceso a recursos financieros para apoyar su/s proyecto/s**  Access to financial resources to support your/s project/s |  |  |
| **Desarrollo de competencias emprendedoras (liderazgo, resolución de problemas, organización, planificación, toma de decisiones, entre otras) para su/s proyecto/s**  Development of entrepreneurial skills (leadership, problem solving, organization, planning, decision making, among others) for your/s project/s |  |  |
| **Influencia recibida por parte de emprendedores/intraemprendedores académicos**  Influence from academic entrepreneurs/intrapreneurs |  |  |
